# Traditional, religious, and cultural perspectives on mental illness: a qualitative study on causal beliefs and treatment use

**DOI:** 10.1080/17482631.2022.2123090

**Published:** 2022-09-13

**Authors:** Muhammad Arsyad Subu, Dave Holmes, Ashokan Arumugam, Nabeel Al-Yateem, Jacqueline Maria Dias, Syed Azizur Rahman, Imam Waluyo, Fatma Refaat Ahmed, Mini Sara Abraham

**Affiliations:** a College of Health Sciences, University of Sharjah, Sharjah, United Arab Emirates; b Adjunct Faculty, Universitas Binawan, Jakarta, Indonesia; c Faculty of Health Sciences, University of Ottawa, Ottawa, IL, Canada; d Politeknik Yayasan Cahaya Padmakumara, Jakarta, Indonesia

**Keywords:** Traditional, alternative, cultural perspectives, mental illness, causal beliefs, treatment use, content analysis

## Abstract

**Purpose:**

Limited information is known from studies regarding traditional, religious, and cultural perspectives on mental illness and the use of traditional and alternative therapies by mentally ill people in Indonesia. This study explored traditional, religious, and cultural beliefs about causes of mental illness and the use of traditional/alternative treatments for mentally ill patients.

**Method:**

We adopted a qualitative content analysis method as proposed by Schreier. This study was conducted at a mental Hospital in Indonesia. We interviewed 15 nurses and 15 patients. Data were analysed using qualitative content analysis method.

**Results:**

Five discrete but interrelated themes emerged: 1) Possessed illness and belief in supernatural forces; 2) Sinful or cursed illness; 3) Witchcraft or human-made illness; 4) traditional/alternative treatments; and 5) Barriers to treatment of mental illness.

**Conclusion:**

Traditional/alternative treatments play an important role in meeting the need for mental health treatment. The findings are relevant for mental health nurses who provide direct to their patients, and for other areas of mental health practice. We also found a lack of knowledge about the causes of mental illness among patients and families. Education should be at the heart of mental health promotion to raise the level of mental health literacy in Indonesia.

## Introduction

The term “mental illness” is used to describe the problems and behaviours related to mental distress included in the International Statistical Classification of Diseases and Related Health Problems, Tenth Revision (World Health Organization, 2013). Globally, mental disorders are of major concern. Approximately 450 million people suffer from these disorders worldwide, with more than 75% of those affected being from developing countries (WHO, 2018). In Indonesia, the reported prevalence of severe mental illness is 1.7 per 1,000 people and about 6% of the total population has a mild mental illness (Ministry of Health of Indonesia, 2013).

During the middle ages, belief in demonic possession was an explanation for erratic behaviour accepted by society (Forcén & Forcen, 2014). Throughout the world, many cultures, societies, and religious groups continue to believe in magic and supernatural phenomenon. Demonic possession refers to the belief that people are possessed by malevolent preternatural beings such as demons (Sarah, 2004). For example, Through the medieval and early modern periods, beliefs in demonic possession as a cause of mental disease have been recorded in Western Europe (Kemp & Williams, 1987). There are also widespread religious beliefs in evil spirits (Yildiz et al., 2010). Some Southeast Asian cultures believe that supernatural powers are responsible for mental illness and view it as the outcome of disrespect for spirits or Gods (Khan et al., 2011; Mishra et al., 2009). In Indonesian traditional culture and society, people believe in various creatures, such as hantu, iblis, jinn, Satan, ruh, and jaelangkung or spirits. Often, Iblis (considered the leader of evil spirits) is claimed to tempt humans to commit sins (Sells, 1995; Subu, 2015). Worldwide, studies have showed that many people believe mental illnesses are “spiritual illnesses” caused by demonic or spirit possessions, witchcraft, or the effect of the evil-eye (Karanci, 2014; Ross et al., 2013; Stefanovics et al., 2016; Tajima-Pozo et al., 2011). The notion of jinn possessing humans is also widespread among Muslims and accepted by most Islamic scholars, even though it is not directly attested in the Quran (Dein & Illaiee, 2013).

In developing countries, practitioners of traditional/alternative (T/A) treatments fill a major gap in mental health service delivery because of beliefs about the causes of mental illness such as those described above (Sherra et al., 2017). For example, the belief that mental illness results from possession by demons means the expulsion of demons is an appropriate treatment (Mercer, 2013). However, some researchers and clinicians have expressed concern that belief in demonic possession may limit access to healthcare for patients with mental illness (Karanci, 2014). Traditional/alternative treatment has a long history (Putro, 2018). This treatment is non-medical treatment where the equipment and materials used are not included in the standard medical treatment and it is not carried out by healthcare professionals (Fanani & Dewi, 2014). Basically, an alternative treatment is not a faith-based treatment (Andira & Pudjibudojo, 2020). According to the World Health Organization (2018a), an alternative treatment refers to a wide range of health-care techniques that are not part of a country’s traditional or conventional medicine and are not fully incorporated into the prevailing healthcare system. In some Asian countries such as Indonesia, China, Korea, and Vietnam, traditional/alternative treatments are officially recognized and incorporated into all aspects of healthcare provision (Gqaleni et al., 2007).

In particular, many people seeking mental health services in developing countries access shamans and religious experts for various alternative remedies (Burns & Tomita, 2015). In Africa, about half of people affected by mental illness seek therapy from shamans and religious practitioners in the first instance of their illness (Burns & Tomita, 2015). T/A treatments are also widely prevalent in countries such as Jamaica (James & Peltzer, 2011). In Indonesia, healthcare treatments for patients with mental illness are not optimal (Maramis, 2007). Minas and Diatri (2008) noted that the quality of mental health services in hospitals is so poor that families tend to bring those experiencing mental illness to religious leaders, shamans or seek alternative therapies from “smart people” or traditional healers Subu et al., 2021b). The Ministry of Health of Indonesia (2003) (cf. No. 1076/Menkes/SK/VII/2003) has issued rules relating to traditional therapeutic practices. However, a review of the relevant literature showed that little information based on scholarly evidence is available related to beliefs about the causes of mental illness and T/A treatments among patients with mental illness in Indonesia. Therefore, this study explored patients’ and nurses’ beliefs about the causes of mental illness and patients’ use of T/A treatments. This study also aimed to summarize knowledge about shared experiences, barriers, and facilitators to treatments of patients with mental illness in Indonesian healthcare settings.

People with mental illnesses in Indonesia are treated utilizing a bio-medical approach. The biomedical paradigm assumes that mental illnesses are caused by biological abnormalities in the brain and stresses pharmacological treatment to address these abnormalities. For more than a decade, the Indonesian mental healthcare system has been controlled by a biologically-focused approach to mental health science and practice (Irmansyah, 2010).

This qualitative study explored traditional, religious, and cultural beliefs about causes of mental illness and use of traditional/alternative treatments by mentally ill patients in Indonesia. Specifically, we aimed to clarify the social, traditional, and religious representations and responses to mental illness in Indonesian culture. Exploring the mentally ill person’s experience of their mental health condition and mental health treatments allowed us to achieve an in-depth understanding of traditional, religious, and cultural beliefs about causes of mental illness and use of traditional/alternative treatments in this context. It is also important to understand the mentally ill person’s experience of stigmatization related to their mental health condition and mental health treatments and strategies are deployed by people with mental illness to adapt to stigma related to mental health and its treatments. The results of this study therefore build on existing literature and may inform specific and effective interventions related to traditional, religious, and cultural beliefs about causes of mental illness and use of these traditional/alternative treatments for mentally ill patients. Finally, the results of this study may improve our understanding of traditional, religious, and cultural beliefs about causes of mental illness and use of traditional/alternative treatments for mentally ill patients in the wider Asian context.

## Methods

### Design, setting, and participants

In this study, we adopted a qualitative content analysis method as proposed by Schreier (2012). Qualitative content analysis is an interpretive process involving systematic coding and categorization that includes a process of understanding, interpreting, and conceptualizing the meanings of qualitative data (Holloway & Wheeler, 2010).

This study was conducted at a mental Hospital in West Java Island, republic of Indonesia. Thirty participants were recruited for this study, including 15 patients and 15 nurses who worked at the hospital. Qualitative research standards indicate that 12–25 interviewees are adequate to achieve data saturation (Charmaz, 2006). The research presentation was the first point of interaction with the nurse participants. Following this initial meeting, a mutually agreed-upon time for the interviews was set. After consulting with the healthcare team and the hospital management, we confirmed that patient participants would be contacted. All participants provided consent to participate and interviews were conducted in the hospital setting. The sites for the interviews were selected to offer participants privacy and quiet in order to make them felt comfortable. In this study, we only included Indonesian adults (aged 18 years and over). All participants (nurses and patients) were Muslim who worked at the hospital. Patients with severe symptoms of their mental illness were excluded. All interviews took place at the hospital, with patients being interviewed in the morning and nurses being interviewed before or after their clinical shift. Study participants were asked a range of questions throughout the 40–60 minute interviews. No participant received any direct compensation for their participation in this research.

### Data collection

The results of qualitative research rely on interviewers collecting data directly from participants. All study participants completed semi-structured interviews conducted by the first and seventh authors (Dr. MAS and Dr. IW). They are both qualitative researchers and they have experienced in conducting qualitative interviews for more than 15 years. Semi-structured interviews are a well-established method of collecting data in qualitative research to enable positive interactions with participants and enable participants to communicate their ideas, past experiences, attitudes, and beliefs (Richards & Morse, 2007). Each interview lasted 40–60 minutes and was conducted in a quiet environment at the study hospital. The interviews were audiotaped using a tape recorder with participants’ consent. The interview questions explored traditional, cultural, and religious beliefs about causes of mental illness and use of T/A treatments for these disorders. In addition, memos and field notes were recorded during data collection to improve the credibility of the data interpretation through triangulation (Lincoln & Guba, 1985). The field notes also helped to record the interviewer’s observations and reflections on the data.

### Data analysis

Content analysis focuses on the subject, background and explores the differences and similarities between and within different parts of the textual information (Graneheim & Lundman, 2004). This method is also used to examine patterns in communication in a replicable and systematic manner (Bryman, 2012), and to obtain rich and deep information about the phenomenon under study (Speziale et al., 2011). NVivo version 12 (QSR International) was used for data management (coding and analysis of relationships between and within text segments). Open coding was used to conceptualize, define, and develop themes derived from the data. To reach overall understanding and consensus among the research team, the transcripts of the interviews were read several times by all members of research team. Next, relationships among the extracted themes were identified using tables and diagrams that represented conceptual patterns. Responses concerning beliefs about the causes of mental illness and experiences of T/A treatments were extracted from the interview transcripts by the first, second and the third authors. All codes and units that were meaningful in the context of the study were interpreted and compared in terms of their similarities and differences. Final abstraction and classification of meaningful subthemes were discussed and agreed by all team members.

### Ethical considerations

This study was performed in line with the principles of the Declaration of Helsinki. Approval was granted by the Ethics Committee (Approval ID: xxxxx). All participants provided verbal and written consent to participate in the study. In this study, we provided information about the study aims and research method to all participants, along with an informed consent form for completion before each interview. All participants were assured of the anonymity (data were coded without names) and confidentiality (information was not shared to others without their permission) of their data. To ensure confidentiality, each participant was assigned a random, alpha-numeric code to make it impossible for anyone to link a transcript to a particular participant (e.g., P1 for participant number one, P2 for participant number two). The content of all interviews (i.e., tapes, videos, and transcriptions) were analysed by the research team. Data were kept in a secure place, and will be retained for a period of 5 years before being destroyed (i.e., tapes will be demagnetized and transcriptions will be shredded).

## Results

This study explored traditional, cultural, and religious beliefs regarding causes of mental illness, T/A seeking behaviours, and barriers to treatment for individuals with mental illness in Indonesia. Based on our qualitative content analysis, five discrete but interrelated themes emerged from the interviews: 1) Possessed illness and belief in supernatural forces; 2) Sinful or cursed illness; 3) Witchcraft or human-made illness; 4) T/A treatments; and 5) Barriers to treatment of mental illness. Eleven subthemes were identified within those five themes.

## Theme 1: Possessed illness and belief in supernatural forces

Participants indicated that in some traditions, cultures, and religions in Indonesia, people believed in the concept of demonic possession.

### Possession

Some participants noted that people believed that if a person had a mental illness, it was because a demon or Satan had possessed that person. One nurse participant said:

Right, yes, it is true … people with mental illness and their families use traditional or alternative therapies or treatments on many smart people, because they consider mental illness [is] due to being possessed [by a] demon. The disorder is attributed to Satan or demon. That is what I know. (P12)

Similarly, because patients assumed Satan or a demon was the cause of their mental illness, they did not go to healthcare facilities for treatment. Instead, they sought treatment from smart people. A nurse participant described this as follows.

… They chose to have treatment to smart people and alternative treatments because they assume that patients get sick, because they are possessed by a demon or Satan. Therefore, they do not go to the hospital or healthcare facilities. They prefer to have treatment with smart people or traditional healers. (P26)

In addition, participants reported that people in Indonesia believed in Roh, Jinn or evil spirits as causes of mental illness. For example, patients were thought (e.g., by their family members) to suffer from mental illness because they were possessed or influenced by a bad jinn or evil spirit. A nurse participant shared that:

Umm, yes … I often meet the patients who are considered that they have contacted and bumped by evil spirits … because of Roh, Jinn. Yes, like that. Very often, family of patients told me that. Sometimes for the patients who have been hospitalizing here, family members bring or leave ‘water’ from a traditional healer or chaplain. (P7)

## Theme 2: sinful or cursed illness

Many Indonesian believe that there is a connection between sickness and sin—including mental illness. Cursed illness is considered as a cause or bad luck for someone or the mental illness condition. Many participants claimed mental illness was caused by one’s own sins or the sins of family members. If a family member suffered mental illness, it was considered a sign of disgrace because the illness was thought to be associated with a sin in that family. A nurse participant described this belief:

… Yes, it is a sin illness. They believe or assume that mental illness can also be because of his family’s attitudes in the past, his [patient’s] mother or father or … the sin of his grandfather or grandmother. Because of this sin-induced illness, they [patients] are considered sinners. (P16)

In addition, some participants assumed that mental illness was a cursed illness. Therefore, families were ashamed of having a family member who suffered from mental illness. Another nurse shared that:

Hmmmm … mental illness is assumed as … is that mental illness is caused by … a cursed illness. Therefore, their families are ashamed. Cursed illness … ’His father has been the same before’ [his father was a person with mental illness]. Like this. Because of the curse[d] illness, they [patients] go to the alternatives or outside treatments [some traditional healers]. So many people believe that. (P5)

## Theme 3: witchcraft or human-made illness

Many Indonesian still believe witchcraft or human-made Illness as a cause of illness, especially mental illness. Some study participants reported the belief that they suffered from mental illness because of witchcraft directed at them by other people. One participant said:

… someone has sent witchcraft to me. There is also [an] orange opened. Moreover, he [shaman] said, ‘a friend of mine in school was used chant to me.’ Yes, my friends … Actually, I do not believe that. However, because of my condition, I want to [be] healthy so that I follow it. After arriving home from Bengkulu [a village], there is no result … Very bad and I am crazy again. (P10)

Similarly, another participant shared that:

You know, to me sir … My neighbor was using a magic to me … Yes, he used magic or witchcraft. This is given by his father and his grandfather … This magic is called as ‘Mulut Keampuhan’ [manmade chants with mantra]. I do not like that, he does it to me … Yes, and this magic makes me angry. (P11)

## Theme 4: T/A treatments

### The first choice of treatment

According to study participants, a T/A Treatment is the first choice of mental health treatment. Participants indicated that patients with mental illness were brought to “smart people” who practice traditional or alternative treatments. Very often, these treatments were the first choice for patients and their families. A nurse participant stated:

Mostly, yes … In general, patients with mental illness go everywhere looking for alternative … the family thinks there is no need to go to a mental hospital. They went to the shamans first, to the pastor first, everywhere first … Yes, they go to the alternative [methods] for treatment first. Generally, they go to the hospital as a last resort. (P17)

### Treatment at the Pesantren (Islamic foundation or school)

Treatment at the Pesantren (Islamic foundation or school) is common among people with mental illness in Indonesia. According to some participants, people sought T/A treatments because of stigma about mental health problems. They presumed that if they received alternative therapies, no “label” of mental illness would be imposed on them. One participant indicated that a popular place for alternative treatment was the Islamic foundation or Pesantren.

… Yeah, I go to the alternatives. This alternative is usually … I go to the Pesantren [Islamic foundation or school], or to Islamic spiritual facility, or Islamic smart people [in their language]. Yes, it is traditional treatment. (P24)

### Treatment by Kiyai or smart people

Kiyai or smart people is popular healers among people with mental illness and their families. Participating patients also indicated that they were taken to Kiyai or smart people before coming to the hospital. A participant informed that he was treated by Kiyai (smart people) and recovered, but the illness relapsed again.

Usually, [I] go to the smart people; they are called Kiyai by people here. Yes, that is true. I was brought to a Kiyai, a smart scholar, by my brother. I was brought there for my recovery, but I [the illness] relapsed again. Then, I came here. I am here [hospital] now. (P12)

### Treatment by a chaplain

Treatment by a chaplain is also popular among people with mental illness and their families. Study participants believed that a chaplain was a suitable choice for treating mental illness. One nurse participant described her thoughts as follows.

Smart people are many kinds outside. There are chaplains, Kiyai, dukun [shamans]. Usually, because they are witchcrafted, they go to the chaplain … Many names are in community. An example is ajengan, a traditional smart person, or a traditional chaplain is a smart person, alternative healer in the community. (P26)

### Treatment by a “dukun” (shaman)

Many people with mental illness were brought by their families to a dukun (shaman) for treatment. A patient explained his experience when he went to a shaman for treatment as follows.

… They took me to the shaman. Everyone is crazy to go to shaman. Yes, I also go to the shaman for my healing … With treatment in the shaman, there were chickens ready to be cut. Whatever is requested by the shamans, we follow. But after that, there is no change … (P20)

### Treatment by a paranormal healer

According to participants, paranormal healing was another alternative treatment option for patients suffering from mental illness. One participant said:

Yes, they go to paranormal [healers]. It is true, yes … Yes … They live next to the paranormal place. In the paranormal [healing], the mental illness is given spell therapy. They (sufferers) are unaware of treatment and spells. Many people are right to lose memory … (P27)

### Treatment by a Chinese therapist

Participants also thought that patients with mental illness turned to traditional Chinese medicine (TCM) for treatment. These Chinese therapists used a variety of remedies including herbs, acupuncture, massage, and diet therapy. A participating patients described their experience with TCM:

Yes, it is true. I have been in traditional Chinese treatment too. Many people also go there. Yes, a Chinese therapist used different herbs and acupuncture. Some use massage and diet therapy for their clients. There, I drink [took] Chinese pills … My family brought me there. (P3)

### Lack of knowledge

The majority of nurse participants indicated that patients and family members chose different T/A treatments because they lacked knowledge about mental illness. For example, a nurse said:

Patients and families do not know about mental illness … and the causes of mental illness … They lack knowledge. Therefore, they try to help with treatments with many alternatives. They go to Pesantren, paranormal [healer], shaman or other places. They bring the patient here, bring there, everywhere. Then, finally, the sufferer is brought here [hospital] … (P18)

### Cost associated with traditional treatments

Some participants also noted that T/A treatments sought by patients and families from smart people or others cost large amounts of money. One participant described this point as follows.

They must have treatments to [from] the alternatives, smart people. ‘We are stupid people’, they say. Yes. Therefore, much money has been spent in these smart people before they go to the hospital, or public health center. (P29)

In contrast, other patients felt that a lot of money was needed for treatment in the hospital. Therefore, they first chose to visit smart people for their treatment.

… First, they assume that having treatment in the hospital needs much money. Second, they chose to have [alternative] treatment from smart people because they assume that patients get sick because they are possessed by [a] demon and so on. Therefore, they do not have treatment in the hospital or healthcare facilities, but they have treatment with smart people. (P1)

### Stigma associated with mental illness

According to some nurses, families did not bring family members with mental illness to the hospital because they felt ashamed as mental illness had a “stigma” label. A participant shared that:

There is a stigma of mental illness. Patients and families are ashamed. For that reason, they do not want to go to the hospital … In reality, many societies of Indonesia have stigma of mental health problem. They consider that mental illness is a disgrace for community members, shame; so, they do not need to be involved in community members; it is a disgrace. (P9)

## Discussion

This study explored traditional, cultural, and religious beliefs regarding the causes of mental illness, T/A treatment seeking behaviours, and barriers to treatment of individuals with mental illness in Indonesia. Our findings revealed different beliefs about the causes of mental illness as well as various traditional treatment-seeking behaviours. In Indonesia, persons with mental illness, their relatives, and community members believed in the concept of supernatural forces and possession by demons (i.e., Satan, evil spirit, roh, jinn, or genie) or evil spirits (i.e., malevolent preternatural beings (Sarah, 2004). Consistent with our findings, supernatural beliefs about the cause of mental illness have been reported in previous studies (Kate et al., 2012; Subu, et al., 2021; Thomason, 2008), and demonic possession has been reported in various countries including India (Kulhara et al., 2000), Pakistan (Choudhry & Bokharey, 2013), Ghana (Adjorlolo et al., 2018), Ethiopia (Teferra & Shibre, 2012), and the United Arab Emirates (Sherra et al., 2017).

Such beliefs are influenced by personal experiences, religious and cultural factors (Furnham & Murao, 2000; Furnham & Wong, 2007). A study conducted in India indicated there was a high prevalence of belief in supernatural forces among patients with mental illness (schizophrenia [96.8%], anxiety [40%], and depression [27.3%]) and their relatives (Chakraborty et al., 2013). Further, demonic possession has been claimed to be associated with schizophrenia (Ross et al., 2013; Tajima-Pozo et al., 2011), dissociative disorder, bipolar disorder, and personality disorder (Thomason, 2008). In addition, Hartog and Gow (2005) found that participants in their study endorsed a demonic aetiology for schizophrenia (37.4%) and depression (38.2%). Moreover, in Ethiopia, mental illness among postnatal women was thought to be caused by curses, bewitchment, “exposure to wind,” and subsequent attack by an evil spirit (Teferra & Shibre, 2012). Similarly, religious beliefs in the United Arab Emirates suggest that different causes of mental illness include bad touch, evil-eye, witchcraft, and jinn possession (Sherra et al., 2017). These findings highlight that religious beliefs and values are widely purported to be predictors of the causes and treatments for mental illness (Hartog & Gow, 2005).

Some of our participants indicated that mental illness was caused by the sins of those with mental illness or the sins of their family members. Similar beliefs have been reported in previous research (Lubis et al., 2014), including the presumption that God cursed them to become sick. Therefore, mental illness has been perceived as a disgrace for the entire family because of sinful actions. In addition to beliefs about demonic possession or sinful actions causing mental illness, people in Africa (Sokhela, 2016) believed human-made or witchcraft actions were a cause of mental illness. Witchcraft is also believed to be a superpower in Asia, maleficium in Latin America, and magic in Europe. It is also common for people to think that a change in their body that was not detected by medical examination was a result of “santet” (magic; Anisa et al., 2017).

Our findings revealed that T/A treatments for mental illness were popular among patients, their families, and community members in Indonesia. Because of the prevailing beliefs in supernatural, spiritual or magico-religious causes of mental illness, spiritual healing has been the preferred approach for mental illness (Subu et al., 2021; Tibebe & Tesfay, 2015). For example, when people are unwell, they commonly pursue various treatment avenues, including orthodox hospitals services, prayer camps, herbal therapy, and traditional healing (Opare-Henaku & Utsey, 2017). Consulting spiritual healers or wise men (indigenous healers), practicing prayers, reciting sacred texts, and using holy water are some of the most common treatment options (Choudhry & Bokharey, 2013; Fellmeth et al., 2015; Mjøsund et al., 2015). Sherra et al. (2017) reported that almost 60% of patient populations in the United Arab Emirates had visited faith healers before seeking medical services. According to Grover et al., 2016), people who sought treatment from faith healers in India substantially more frequently attributed their symptoms to supernatural causes. One of the most common T/A treatments described by participants was Pesantren (Islamic foundation or school). According to patients with mental illness, when they sought alternative therapies, no “label” of mental illness was imposed on them; therefore, they went to Pesantren led by Islamic teachers or religious leaders known as “Kiyai” or “Islamic clerics” (Lukens-Bull, 2005; Nelson, 1999). Many Indonesian Muslims think a kiyai is more respected than a ustadz because he runs his own boarding school and has magical abilities (Lukens-Bull, 2005). Because people will listen to what a kiyai says, he has power and influence (Platvoet & van der Toorn, 1995). As spirituality is a key aspect of positive mental health in the local population (Vaingankar et al., 2011), an important factor is including religious leaders in raising mental health literacy. (Bhikha et al., 2015). In Morocco, where the concept of sorcery is blended with Islamic concepts of jinn to explain mental illness, patients are often treated by religious scholars with the dual roles of sorcerer and holy man (Stein, 2000; Subu et al., 2021). People with mental illness in Singapore reported turning to spiritual or religious counsellors or other healers for assistance (Chong et al., 2012). Frank and Frank (1991) noted that one of the roots of modern psychotherapy is spiritual healing, and this tradition remains influential even in secularized Western societies.

Some of our participants also sought treatment by dukun or shamans who conducted various mental health treatments, as these people are easily accessible in the Indonesian context (Widiastutik et al., 2016). Shamans are traditional healers that follow indigenous traditions (Walsh, 2007); they are believed to communicate with both good and evil spirits (Mercer, 2013) and use exorcism as a ritual to drive the devil, demons, or evil spirits from a person (Wilkinson, 2007). People of Indonesia have long believed in supernatural phenomena such as ghosts, spirits, and witchcraft. A dukun is said to have the ability to connect with malicious spirits.

Similar beliefs also exist in many other places, such as Malaysia (Razali et al., 1996) and Kenya (Musyimi et al., 2017). As some participants believed mental illness resulted from witchcraft directed by others, they went to chaplains (“ajengan”) available in Christian communities. In many Christian belief systems, Satan and the devil are described as fallen angels (MacKenzie, 1999). In addition to receiving patient referrals, chaplains also carry out spiritual assessments, lead support groups, and create opportunities for pastoral care (Eagger et al., 2009). According to Hewson (2012), a chaplain can provide spiritual support for service personnel in healthcare settings. All of these functions are delivered by a highly motivated workforce, but one that has little or no specialized training (Gubi & Smart, 2013). Pennybaker et al. (2016) suggested that there is a need to provide chaplains with training in mental healthcare.

Our study findings showed that paranormal healing using prayers was another popular treatment for mental illness in Indonesia. Previous research found that about 46% of people with mental illness in Indonesia sought treatment from paranormal healers or smart people (Keliat et al., 2011). Only a few researches have looked into the effects of paranormal practices, and no experimental results have achieved widespread recognition as genuine proof of the paranormal in the scientific world (Odling-Smee, 2007). Finally, some Indonesian patients with mental illness visit traditional Chinese healers or TCM practitioners. TCM recognizes mental illness as a result of the disequilibrium of yin-yang and a disorder of qi and their viscera (Lam et al., 2016). The practice of TCM in Indonesian society includes various forms of herbal medicines, massage, acupuncture, and diet therapy (Shang et al., 2007). There appears to be some positive impact from TCM practitioners in handling patients with mental illness (Lam et al., 2016), although further exploration of this is needed. Traditional medicine, including that supplied by paranormal and TCM procedures, has been governed by particular standards in Indonesia. The Indonesian Ministry of Health enacted this rule (Ministry of Health of Indonesia, 2003).

## Barriers to using mental health treatments

This study identified some barriers to seeking professional mental health treatments, including lack of knowledge about mental illness, expensiveness of treatment (Saxena et al., 2007). This was consistent with evidence that suggests that there is a low level of mental health literacy in developing countries (Harner, 1990; Tibebe & Tesfay, 2015). Different sociocultural meanings of mental illness and associated beliefs may have an impact on how people use services, decide which treatments and therapies to use, and respond to professional mental healthcare (Corrigan, 2004). Some people may not consider a mental health clinic as an appropriate avenue for treatment as they feel mental health professionals disregard religious values and therefore prefer to approach traditional healers (Al-Krenawi & Graham, 2000; Bhikha et al., 2015). Consistent with our findings, many studies have reported a lack of awareness among patients with mental illness and their family members about various treatment options and their availability (Bowers et al., 2013; Choudhry & Bokharey, 2013; Naeem et al., 2012; Saxena et al., 2007). Further barriers to seeking mental health treatment for those with mental illness include fear, lack of knowledge about the illness, avoidance of symptoms, feeling of shame, and cultural beliefs (Shannon et al., 2015). We also found that considering a label of mental illness as stigmatizing was a barrier to accessing modern mental health facilities and treatment. Mental illness is widely stigmatized in Indonesian society. The majority of mentally ill Indonesians have been stigmatized by the general public, the government, healthcare staff, and the media (Hawari, 2001). Since the stigma of mental illness is rarely discussed openly, this produces misunderstanding among population. Family members often hide the patient with mental illness because they are ashamed to bring them to a psychiatric care setting. If families do take their relatives to a psychiatric hospital, they rarely visit them. This was consistent with previous research that showed that in some cultures, people with mental illness are stigmatized, discriminated against, or marginalized in society, which presents a major obstacle to consulting professional mental healthcare services (Choudhry & Bokharey, 2013; Furnham & Murao, 2000; Liu et al., 2015; Shannon et al., 2015; Wahl, 2012).

Our study results provide useful information to inform further research with people with mental illness concerning the use of T/A therapies in Indonesia. In particular, further research is needed to investigate the effectiveness of T/A treatment because such methods are under-researched and documented in Indonesia. It is also important that research is needed to understand collaboration between traditional healers and mental health professionals in Indonesia. In addition, this study did not focus on the beliefs and perspectives of families, communities, and governments on mental illness and the use of alternative therapies; further studies are needed to expand knowledge on these perspectives. Finally, studies investigating factors affecting the use of T/A treatments by individuals with mental illness in Indonesia are warranted.

## Conclusion

The present study highlighted that supernatural beliefs are common in Indonesian traditions, culture, religions, and society. Discussion of traditional, religious, and cultural perspectives on mental illness is important. Participants in our study attributed the symptoms of mental illness to these beliefs. Our findings offer some understanding about traditional, religious, and cultural viewpoints of mental illness, and there is potential for these findings to be translated to the Indonesian practice context. The applicability of the present study is supported by its epistemology. For example, social constructionism contends that cultural interactions shape reality and that a history of shared values or traditions is crucial for mental health (Burr, 2015; Willig, 2013). The idea of cultural identity put forward by Collier and Thomas (1988) suggests that common cultural beliefs, norms, values, and meanings influence how people behave in certain situations. This incorporates components of cultural ideals, beliefs, or propensities for action that are supported by an ethnic population (Unger, 2011). Over the course of a person’s lifetime, cultural identification is a fluid and dynamic process that constantly changes over the course of a person’s lifetime (Bauman, 2001). Importantly, cultural values, beliefs, and customs have a significant impact on how people think and behave when seeking mental health care (Jidong et al., 2021; Makgahlela et al., 2021).

Our study showed that T/A treatment methods played an important role in meeting the need for mental health treatment in the Indonesian population. Many Indonesians believe that patients with mental illness are possessed by devils, demons, or spirits, or that they are being punished for evil deeds or the use of illegal psychoactive substances (Audu et al., 2013).

Our study showed that many patients in clinics and hospitals had first consulted one or more traditional healers. Alternative treatments and smart people were the primary option for patients with mental health problems and their families. Traditional healers or “wise people” available to patients or their families in Indonesia include Islamic schools (Pesantren) led by Islamic professors or religious leaders (known as Kyai or ulama), Christian chaplains, dukuns or shamans, individuals practicing traditional Chinese medicine, and paranormal healers. These treatment methods offered by Islamic religious scholars (Kyai), paranormal healers, chaplains or pastors, TCM practitioners, and shamans are often the first choice for patients with mental illness and their families in Indonesia. Our findings offer important information for mental health nurses who provide direct nursing care to their patients, as well as for other areas of mental health practice.

Implications for mental health practice and education

Our findings are particularly relevant for mental health nurses who provide with hands-on nursing care for patients. It is important to properly support mental health nurses who interact with their patients and improve mental health education to help minimize some of the negative effects of poor mental health literacy or lack of information. This will also empower and educate individuals, families, and communities. We found that a major barrier to treatment was the lack of knowledge and information. Interventions that aim to increase public understanding of the causes and treatment of mental illness may reduce the time it takes for someone to seek treatment and enhance the effectiveness of that treatment. It is essential that people receive education to help them recognize the signs and symptoms of mental illness as soon as possible because early intervention is necessary to restore a person’s mental, physical, and social health.

In Indonesia, there is a paucity of knowledge and instruction about mental health problems. Therefore, public resources and education are crucial. Our findings offer some practical resources and information that can be implemented in nursing and other mental health education to aid existing and upcoming providers of mental health services in Indonesia to better assist those who are suffering from mental illness.

Recommendations

In some parts of Indonesia, mental health services remain unavailable. It is imperative that mental health organizations operating in Indonesia take immediate action to address this problem. We recommend decentralizing mental health services from urban areas to rural health facilities to support broader provision of mental health treatment. In addition, there is a need to specifically prepare mental health professionals to work in rural settings. This will foster an environment where these mental health professionals can more effectively help their patients. Knowledge and attitudes about mental illness that support early detection, treatment, and prevention are referred to as mental health literacy. Patients, families, and the general public need to be educated about mental illness to improve their health literacy. Importantly, people need to be aware of the causes of mental illness and where to seek for support.

Our study highlighted a lack of knowledge about the causes of mental illness among people with mental illness and their families. Coupled with widespread social stigma, this presented an important barrier for seeking appropriate treatment from mental health professionals. Therefore, education and communication should be central to mental health promotion activities to increase the level of mental health literacy in Indonesia.

We also found there was a lack of support for people with mental illness from their family members. Many families affected by mental illness appeared to feel as if society looked down on them, which created additional psychological strain and may also negatively affect family members’ efforts to assist relatives with mental illness in their recovery. In addition, we found that there was a lack of support from society. To overcome barriers associated with social perception and enhance the level of acceptance of people with mental illness, we recommend that society, families, and peers are educated about the need to provide a supportive environment. Finally, it is not possible to generalize our findings. Using only one mental health facility reflected a sample of Indonesian nurses, not the entire population of mental health nurses. Further studies conducted in different mental health settings with different samples could provide additional useful information.

## Limitations

This study has limitations. The nature and size of the sample in this study meant that analysis was focused on individual perceptions of traditional, religious, and cultural on mental illness, rather than providing a broader social–structural analysis. Given that the study only included 30 participants, these findings cannot be assumed to be representative of all Indonesians population. The information acquired was unique to each of the participants’ opinions and experiences, and as a result, it cannot be applied to the overall nurses and mentally ill population. Generalization is also difficult because data were collected from one setting with the same religion (Muslim) of participants. Therefore, study findings may not be applicable for different Indonesian populations such as Bali, Flores, Kupang, and Manado.

## Data Availability

The data sets for this study can be made available upon request to the Principal Investigator and according to the ethical approval.
